# Extraction and Identification of Volatile Organic Compounds in Scentless Flowers of 14 *Tillandsia* Species Using HS-SPME/GC-MS

**DOI:** 10.3390/metabo12070628

**Published:** 2022-07-08

**Authors:** Alexandre Gonzalez, Zohra Benfodda, David Bénimélis, Jean-Xavier Fontaine, Roland Molinié, Patrick Meffre

**Affiliations:** 1 UPR CHROME, Université de Nîmes, CEDEX 1, 30021 Nîmes, France; alexandre.gonzalez@unimes.fr (A.G.); zohra.benfodda@unimes.fr (Z.B.); david.benimelis@unimes.fr (D.B.); 2 UMR INRAE 1158 Transfrontalière BioEcoAgro, BIOlogie des Plantes et Innovation (BIOPI), UPJV, UFR de Pharmacie, 80037 Amiens, France; jean-xavier.fontaine@u-picardie.fr (J.-X.F.); roland.molinie@u-picardie.fr (R.M.)

**Keywords:** *Tillandsia*, headspace solid phase microextraction (HS-SPME), gas chromatography-mass spectrometry (GC-MS), scentless flowers, faint-scented flowers, volatile organic compounds (VOCs), PCA analysis, heatmap

## Abstract

VOCs emitted by flowers play an important role in plant ecology. In the past few years, the *Tillandsia* genus has been scarcely studied according to the VOCs emitted by flowers. Hence, we decided to enlarge the VOCs composition study already undergone in our laboratory on fragrant 3 *Tillandsia* species to 12 unscented and 2 faint-scented *Tillandsia* species and hybrids. The headspace solid phase microextraction (HS-SPME) coupled with gas chromatography combined with the mass spectrometry (GC-MS) method was used to explore the chemical diversity of the VOCs. This study allowed the identification of 65 VOCs among the 14 species and between 6 to 25 compounds were identified in each of the species. The aromatic profile of 10 of the species and hybrids are similar to each other’s and show 8 predominant compounds: benzaldehyde, benzacetaldehyde, hexanol, hexanal, heptanal, octanal, nonanal, and furan-2-pentyl. Some specific compounds are present only in some unique species such as trans-calamenene, α-muurolene, and α-guaiene trans-β-bergamotene. The two faint-scented species studied present an original aromatic profile with a high number of monoterpenes or phenylpropanoids/benzenoids. Our studies allow a better understanding of the ecological role and function of these VOCs in the interactions between these plants with their environment.

## 1. Introduction

Most *Tillandsia* are epiphytic plants that are distributed from the South of the United States to the South of Argentina and grow by up-taking nutrients present in the air and rainwater [1]. The *Tillandsia genus* belongs to the Bromeliaceae family [2,3,4], and is considered to be the most diverse genus with more than 700 *Tillandsia* species identified. [1,5,6]. The *Tillandsia* genus is well described for its medicinal applications [1,6,7] and its pollution bioindicator potential [8,9,10], but there is a lack of examples dealing with its aromatic profile. Some studies about the phytochemistry of *Tillandsia* species have been reported. Flavonoids, triterpenoids, sterols, and phenylpropanoids are chemical constituents in those species and are well known for their biological activities [1].

Volatile Organic Compounds (VOCs) are often associated with plants fragrance, especially flowers. Fragrant plants produce and emit many different families of VOCs. More than 1700 volatile compounds have been identified and can be classified in the following categories: terpenoids, phenylpropanoids, fatty acid, and amino acid derivatives, [2,3]. All these classes of compounds possess their own biosynthetic pathways such as the acetate pathway for fatty acids derivatives or the mevalonic acid pathway for sesquiterpenoids. For example, phenylpropanoids, benzenoids and their derivatives are biosynthesized through the shikimate pathway [11]. Generally, the VOCs emitted by flower plants have a low molecular weight [12]. Concerning the non-fragrant plants, some species can be scentless for human beings while other species might have a subtle scent only detected by some people. Non-fragrant flowers also emit different VOCs such as aliphatic aldehydes, aliphatic alcohols, and phenylpropanoids/benzenoids [13,14]. VOCs’ role in plants is essential and varied from pollinator attraction to defense against predators and growth [4,15]. Plants, through the VOCs emitted by flowers, are in constant interaction with their environment such as micro-organisms, herbivorous insects, pollinators, or plants between themselves [11].

It has been shown that comparable visual and olfactory signals were due to the plant pollination by close pollinators [16]. However, in a mix composed of major and minor VOCs, the most abundant compounds do not determine the trend of the response [11]. Studies have shown that VOCs of non-fragrant flowers also attract pollinators [17,18]. Moreover, recent studies indicate that scentless Australian orchids emit volatile organic compounds highly attractive to the male wasp [19,20]. A study on *Tillandsia macropetala* was carried out in 2014 by Aguilar-Rodriguez et al. and showed a correlation between the presence of volatile compounds and the pollination of bats [21]. Nevertheless, *T. macropetala* has been removed from the *Tillandsia* genus in 2016. Finally, the role of floral scent not has only an ecological role, but also an aesthetic character of ornamental plants [22].

Gas chromatography combined with a mass spectrometer (GC-MS) is the most commonly used technique to identify VOCs in flowers. To isolate them, different techniques can be used, each one with their advantages and drawbacks such as large quantities of solvent used [15]. The steam distillation, the solvent extraction, and the headspace solid phase microextraction (HS-SPME) are the most common techniques to extract and isolate volatile compounds from a plant [23]. Nowadays, HS-SPME is widely used to extract and identify volatile compounds from flowers [24]. It allows the extraction and introduction of analytes into the GC inlet in one step. It is a rapid technique, which prevents the use of organic solvents and is characterized by a reduced operating cost due to the reuse of extraction fibers [25].

In a recent study conducted in our laboratory by Mame Lo et al., a HS-SPME/GC-MS method was developed to study the VOCs emitted by the fragrant flowers of three *Tillandsia* species. First, two extraction methods were developed and allowed the identification of 30 volatile compounds from *T. xiphioides* [26]. Then, these two methods were used to study the aromatic profile of *T. caliginosa* and *T. crocata* and allowed to identify 65 compounds [27]. Before this work, only one pioneering study about the aromatic profile of the *Tillandsia* genus was carried out in 1991 for *T. crocata* species [28]. This led to a partial isolation of VOCs (seven volatile compounds) from its flowers, as part of a larger study on fragrance composition of flowers attracting male euglossine bees.

For the present study, the two extraction methods developed in our laboratory by Mame Lo et al. [26] were used to obtain the aromatic profiles of 14 species and hybrids of *Tillandsia* genus, belonging to a different subgenus [5]. These species are: *T. aeranthos*, *T. bergeri*, *T. albertiana*, *T. ixioides*, *T. ionantha*, *T. tenuifolia*, *T. paleacea*, *T. cacticola*, *T. lorentziana*, *T. didisticha*, *T. bandensis*, *T. recurvata*, hybrids of *T. aeranthos* and *T. bergeri (T. aeranthos* x *T. bergeri*), and *T. aeranthos* ‘*uruguay*’, a form of *T. aeranthos* (see Section 4.1). All the 14 species have scentless or faint-scented flower odors for humans. However, *T. bandensis* and *T. paleacea* are considered to be faint-scented flowers for some people, while the 12 other species are considered scentless for humans. The objective of this study is to identify the VOCs of non-fragrant flowers of these 14 species and hybrids of the genus *Tillandsia* using the HS-SPME/GC-MS method. The final objective is to confirm that non-fragrant *Tillandsia* flowers also emit common VOCs. All the flowers are presented in Table 1.

A qualitative heatmap was performed to identify in which species the different compounds were present. Then, a principal component analysis (PCA) was performed to profile the variations of compounds of two different species and their hybrids, *T. aeranthos*, *T. bergeri,* and *T. aeranthos* x *T. bergeri* (hybrids). Finally, another PCA analysis was performed including most of the *Tillandsia* species studied in order to try to classify *Tillandsia* species into groups corresponding to the subgenus groups, if possible. The aromatic profile of these *Tillandsia* species has never been studied before and it is the first time an analysis and comparison of the profiles of volatile compounds between non-fragrant *Tillandsia* species and fragrant ones has been realized.

## 2. Results

### 2.1. Selection of the SPME Fiber

For this study, three different fibers were tested on *T. bergeri* flowers to select the more efficient one. The three fibers were tested successively on the two *T. bergeri* flowers each time. The first extraction method was performed at 30 °C for 20 min. Two compounds were extracted for the Carboxen/Polydimethylsiloxane (CAR/PDMS) fiber, one compound for the Carboxen/Polydimethylsiloxane/Divinylbenzene (CAR/PDMS/DVB) fiber, and zero compound for the Polydimethylsiloxane (PDMS) fiber. The second extraction method at 75 °C for 65 min allowed the extraction of 10 compounds with the CAR/PDMS fiber, 6 compounds with the CAR/PDMS/DVB fiber, and 2 compounds with the PDMS fiber. This fiber screening showed that the CAR/PDMS fiber seemed to be the most appropriate fiber for this study due to its specificity [29,30]. Consequently, the CAR/PDMS fiber was chosen for the whole study.

### 2.2. Identification

The CAR/PDMS fiber was used with the extraction conditions developed in our previous studies for the identification and the extraction of volatile compounds from flowers of the 14 *Tillandsia* species [26,27]. Using the HS-SPME/GC-MS technique, a total of 65 compounds were identified from the floral emissions of *Tillandsia*, 12 monoterpenes, 9 sesquiterpenes, 17 phenylpropanoids/benzenoids, 5 aliphatic alcohols, 13 aliphatic aldehydes, and 9 other compounds such as furan-2-pentyl, 2 ionones, and 2 esters, for instance (see Table 2). Among these volatile compounds, 8 (benzaldehyde (22), benzacetaldehyde (24), hexanol (40), hexanal (43), heptanal (44), octanal (47), nonanal (49), and furan-2-pentyl (59)) are present in at least 60% of the 14 species. Qualitative and quantitative differences on the aromatic profile were observed and are presented in the Table 2.

For the 10 species *T. aeranthos*, *T. bergeri*, *T. aeranthos x T. bergeri*, *T. aeranthos ‘uruguay’*, *T. albertiana*, *T. ixioides*, *T. ionantha*, *T. tenuifolia*, *T. cacticola,* and *T. lorentziana*, their aromatic profiles are quite similar between each other’s and are largely dominated by the 8 volatiles compounds described just above: benzaldehyde (22), benzacetaldehyde (24), hexanol (40), hexanal (43), heptanal (44), octanal (47), nonanal (49), and furan-2-pentyl (59). The number of volatile compounds extracted for each species is comprised between 8 (*T. tenuifolia*) to 25 compounds (*T. cacticola*) when both extraction methods are combined. The main difference can be observed with some specific compounds that are only present in one species such as sesquiterpenes trans-calamenene (21) identified in *T. aeranthos*, α-muurolene (19) identified in T. *aeranthos ‘uruguay’* and α-guaiene (15) identified in *T. albertiana*.

For *T. paleacea*, a total of 25 compounds were identified. The eight compounds described earlier are also present. Nevertheless, this species presents a large variety of volatile molecules with seven monoterpenes and eight phenylpropanoids/benzenoids.

For *T. bandensis*, a total of 15 compounds have been identified. *T. bandensis* presents an original aromatic profile. For instance, it is the only species in which hexadecanol (42), tetradecanal (54), and hexadecanal (55) have been identified for the aliphatic alcohols and aldehydes. Furthermore, 4 monoterpenes were extracted, isomyocorene (7), β-ocimene (9), γ-terpinene (10), and terpinolene (11). It is the second species after *T. paleacea* with a high number of identified monoterpenes. Finally, it is the only species with the phenylpropanoids/benzenoids methyl ortho-anisate (35) and phenylethyl acetate (29).

For *T. didisticha*, only six compounds were identified in the floral emission with the two extraction methods, benzaldehyde (22), benzacetaldehyde (24), 2-phenylethanol (26), 2-methoxy-4-vinylphenol (31), hexanol (40), and nonanal (49).

Finally, for *T. recurvata*, a total of 15 compounds were identified in the floral emission. It is the only species with trans-β-bergamotene (20) in the aromatic profile.

### 2.3. Chemometric Analysis of Volatile Organic Compounds

**Figure 1:**
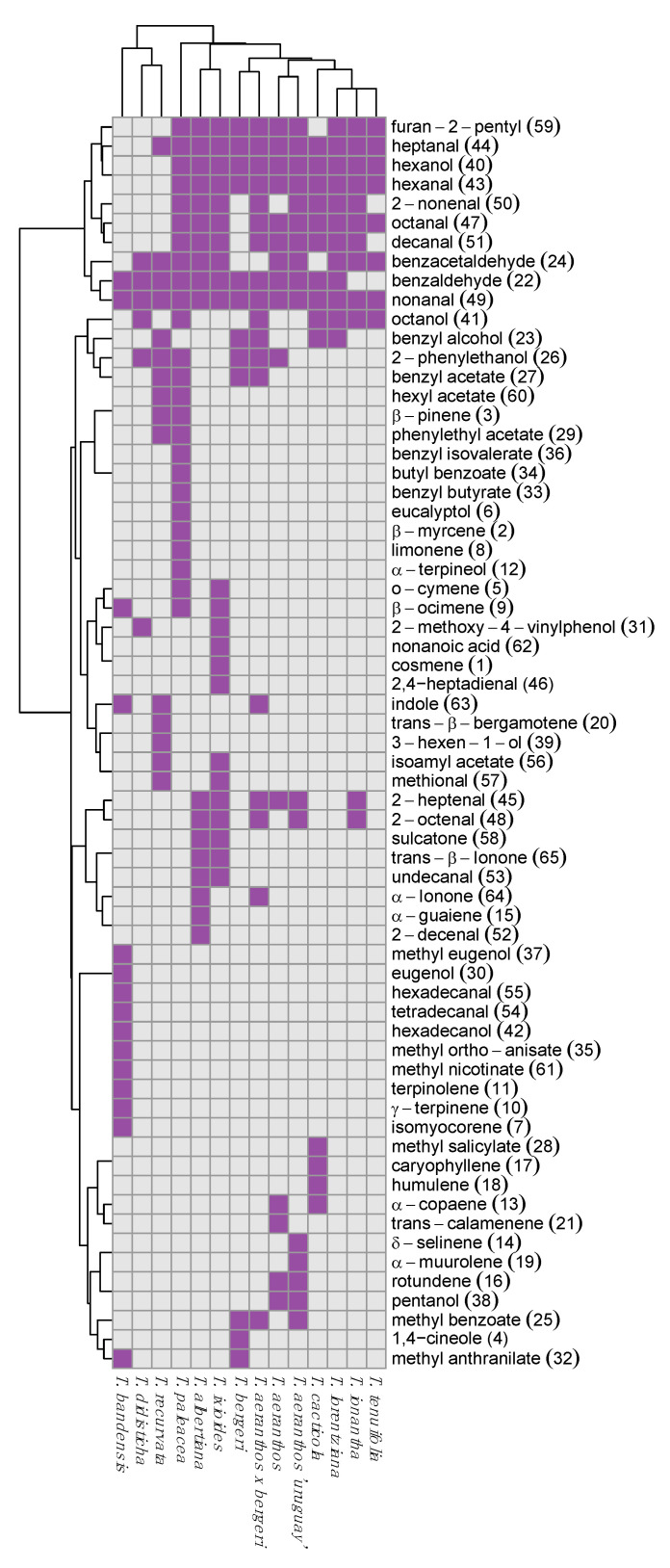
Heatmap from the 14 flowers, in which VOCs were identified or not. Purple represents the volatile organic compounds, which were identified in the corresponding Tillandsia species, while grey represents the non-identified compounds.

A qualitative heatmap was performed considering only the presence or absence of the volatile organic compounds into the different species (Figure 1). The qualitative heatmap’s objective was to observe the specificities and similarities of each species concerning their aromatic profile composition. The main aim was to easily see the composition of VOCs of each species and evaluate the main differences. Indeed, the 14 *Tillandsia* species are grouped into 4 groups in the dendrogram: the first one, with *T. aeranthos*, *T. bergeri*, *T. aeranthos* x *T. bergeri*, *T. aeranthos ‘uruguay’*, *T. ionantha*, *T. tenuifolia*, *T. cacticola*, *T. albertiana*, *T. ixioides* and *T. lorentziana*.; the second one, with *T. paleacea*; the third one, with *T. recurvata* and *T. didisticha’* and the fourth one, with *T. bandensis*. Regarding the heatmap, the compounds that are specific of a species can be easily identified. Indeed, for *T. bandensis*, 10 compounds are only present in this species: methyleugenol (37), eugenol (30), hexadecanal (55), tetradecanal (54), hexadecanol (42), methyl ortho-anisate (35), methyl nicotinate (61), terpinolene (11), γ-terpinene (10), and isomyocorene (7). Likewise, *T. paleacea* has seven compounds specific to its flowers: benzyl isovalerate (36), butyl benzoate (34), benzyl butyrate (33), eucalyptol (6), β-myrcene (2), limonene (8), and α-terpineol (12). Moreover, this qualitative heatmap allows for the identification of the main compounds present in most of the species studied. For instance, as explained above, benzaldehyde (22), benzacetaldehyde (24), hexanol (40), hexanal (43), heptanal (44), octanal (47), nonanal (49), and furan-2-pentyl (59) are the 8 compounds most represented in the 14 species of *Tillandsia* studied (top right corner with green cells in the heatmap). Furthermore, this heatmap allows identifying the *Tillandsia* species with only few VOCs extracted from flowers such as *T. didisticha*.

To help with the identification of the volatile compounds that allow us to highlight similarities or differences between the 14 different species, a principal component analysis (PCA) was used to analyze the 65 compounds identified in this study.

**Figure 2:**
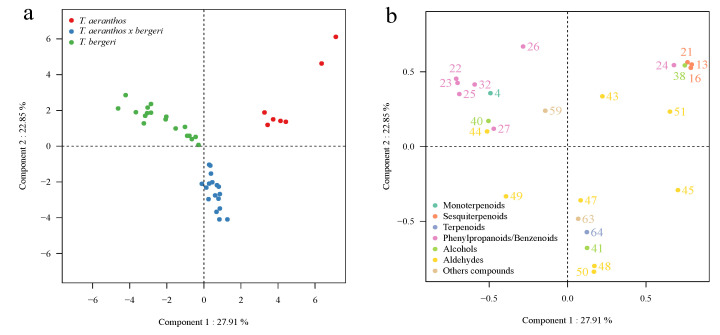
PCA based on VOCs emitted by *T. aeranthos*, *T. bergeri,* and *T. aeranthos* x *T. bergeri* (hybrids of the two species). (**a**) Score plot of PC1 scores versus PC2 scores. (**b**) Loading plot of PC1 and PC2 contributing volatile compounds. Numbers in (**b**) correspond to the compounds numbers listed in Table 2.

Two PCA were realized. The first one corresponds to two *Tillandsia* species: *T. aeranthos* and *T. bergeri* and their hybrids: *T. aeranthos x T. bergeri*. As these species are widely cultivated in the *Tillandsia* PROD plant nursery and are well acclimated to the Occitanie region, it is interesting to study them apart from the 11 other *Tillandsia* species. Moreover, it is very valuable to discover the similarities and differences between the two botanical species *T. aeranthos* and *T. bergeri* and their hybrids, *T. aeranthos* x *T. bergeri*. In Figure 2, the first principal component (PC1) contributes to 27.91%, and the second principal component (PC2) contributes to 22.85% of the total variance in the volatile peak area data. Three distinct groups are identified: *T. aeranthos* flowers are clustered together (in red), same for *T. bergeri* flowers (in green) and the hybrids *T. aeranthos* x *T. bergeri* flowers (in blue). This shows a high degree of clustering of flowers belonging to the same species, thus suggesting a unique volatile profile for each of these three species and hybrids of *Tillandsia*. PC1 allows the partition of *T. aeranthos* flowers from *T. bergeri* and *T. aeranthos x T. bergeri* flowers. This separation is due to the presence of sesquiterpenes in *T. aeranthos* flowers contrary to *T. bergeri* and the hybrids *T. aeranthos* x *T. bergeri.* The higher presence of phenylpropanoids/benzenoids in the hybrids *T. aeranthos* x *T. bergeri* and *T. bergeri* flowers allows the separation with *T. aeranthos* (Figure 2). PC2 shows the difference of the floral emission on one side *T. aeranthos* x *T. bergeri,* and on the other side *T. bergeri* and *T. aeranthos*. This can be explained by the more intense presence of phenylpropanoids/benzenoids in *T. bergeri* and *T. aeranthos* rather than in *T. aeranthos* x *T. bergeri*. In the same way, one monoterpenoid has been identified in *T. bergeri* and the sesquiterpenoids were only present in *T. aeranthos,* which increases the separation through component 2 of *T. aeranthos* x *T. bergeri* on one side with *T. aeranthos* and *T. bergeri* on the other side. 

**Figure 3:**
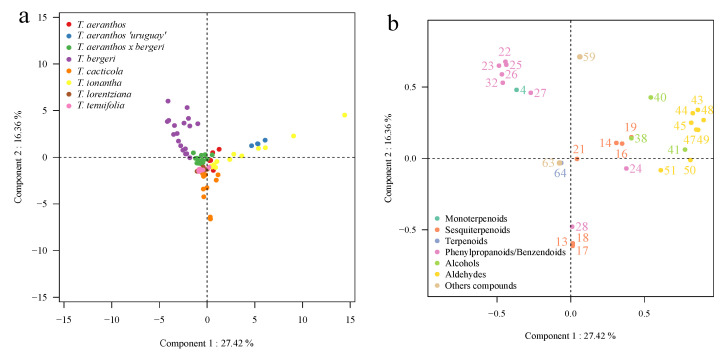
PCA based on VOCs emitted by *T. aeranthos*, *T. bergeri*, *T. aeranthos* x *T. bergeri* (the hybridization of the two species), *T. aeranthos* ‘*uruguay*’, *T. cacticola*, *T. ionantha*, *T. lorentziana*, and *T. tenuifolia*. (**a**) Score plot of PC1 scores versus PC2 scores. (**b**) Loading plot of PC1 and PC2 contributing volatile compounds. Numbers in (**b**) correspond to the compounds numbers listed in Table 2.

The second one is a PCA of eight species: *T. aeranthos*, *T. bergeri*, *T. aeranthos x T. bergeri*, *T. aeranthos ‘uruguay’*, *T. ionantha*, *T. tenuifolia*, *T. lorentziana,* and T. *cacticola*. As described above using the heatmap (Figure 1), *T. paleacea*, *T. bandensis*, *T. recurvata,* and *T. didisticha* have an aromatic profile too different from the other species to be studied in the same PCA. Indeed, *T. paleacea* and *T. bandensis* should be analyzed alone. In Figure 3, the first principal component (PC1) contributes to 27.42%, and the second principal component (PC2) contributes to 16.36% of the total variance in the floral volatile peak area data. Compared to the first PCA described just above (Figure 2), in this PCA (Figure 3), the separation of the eight different *Tillandsia* species is clearly less efficient. A high degree of clustering of flowers belonging to the same *Tillandsia* species is shown in this PCA. Thus, the hypothesis of a unique volatile profile for each species studied can be made. PC1 allows the partition of *T. bergeri* in one side and *T. aeranthos ‘uruguay’* and *T. ionantha* on the other side. The other species are quite clustered in the center of the PCA. PC2 allows the partition of *T. cacticola* from the clustered species and of *T. bergeri* from the clustered species. Consequently, *T. bergeri* and *T. cacticola* are well separated thanks to PC2. As previously described, *T. bergeri* is a clearly distinguish species with the more intense presence of phenylpropanoids/benzenoids. The presence of sesquiterpenoids for *T*. *aeranthos ‘uruguay’* and aldehydes in *T. ionantha* can explain the good separation according to PC1. 

## 3. Discussion

A HS-SPME/GC-MS technique was used to identify the volatile compounds of 14 unscented or faint-scented flowers of species and hybrids of the *Tillandsia* genus. For the extraction using the HS-SPME technique, a CAR/PDMS fiber was chosen and performed with two different extraction methods differenced by time and temperature values, which allowed an efficient extraction of the aromatic components. Indeed, two other fibers were tested—PDMS and CAR/PDMS/DVB. The CAR/PDMS fiber proved to be more efficient by extracting the maximum of compounds in *T. bergeri* flowers. Considering the volatility of analytes affecting the optimal conditions of temperature and extraction time, two extraction methods were necessary to extract the majority of volatiles emitted by *Tillandsia* flowers [26]. The aromatic profiles of the 14 species were obtained by GC-MS analysis. First, the results showed that the number of volatile compounds present in each profile is quite low. Between 6 and 25 compounds per analyzed species were identified in the 14 profiles. In the precedent study of our laboratory, using the same extraction conditions, 30 compounds were identified in the fragrant flowers of *T. xiphioides* [26], 47 compounds in *T. crocata,* and 43 compounds in *T. caliginosa* [27].

These results now reported showed some differences between the profiles of the unscented or faint-scented species studied here. Indeed, *T. paleacea* and *T. bandensis* are two species that are very different from the others. Their aromatic profiles have higher intensities in monoterpenes compared to the other flowers’ species. This can be explained as these two flowers are considered more odorant compared to the 12 other species that are scentless for humans [31,32]. This is confirmed by the interpretation of the heatmap (Figure 1), where it is possible to see the specific compounds identified in both T. *paleacea* and T. *bandensis*. Although these two species are both faint-scented to humans, they present a different aromatic profile between each other. In *T. paleacea*, as the other species, the eight volatile compounds, benzaldehyde (22), benzacetaldehyde (24), hexanol (40), hexanal (43), heptanal (44), octanal (47), nonanal (49), and furan-2-pentyl (59) were identified whereas only benzaldehyde (22) and nonanal (49) where identified in *T. bandensis*. Moreover, 10 compounds were identified only in *T. bandensis*, methyl-eugenol (37), eugenol (30), tetradecanal (54), hexadecanal (55), hexadecanol (42), methyl ortho-anisate (35), methyl-nicotinate (61), terpinolene (11), γ-terpinene (10), and isomyocorene (7). On the contrary, *T. didisticha*’s aromatic profile is very poor. With the two methods, only six compounds were extracted and identified in this species. By regarding the first PCA (Figure 2), *T. aeranthos*, *T. bergeri* and hybrids *T. aeranthos x T. bergeri* can be well separated, which means their aromatic profile is distinctly different between each other’s. Indeed, sesquiterpenoids are specific to *T. aeranthos* while phenylpropanoids/benzenoids are specific to *T. bergeri*. Concerning the second PCA (Figure 3) in which *T. paleacea*, *T. bandensis*, *T. didisticha*, *T. recurvata*, *T. albertiana,* and *T. ixioides* were excluded, the separation of the eight remaining species is more difficult than previously thought. The aromatic profiles of these eight *Tillandsia* species and hybrids share more similarities to each other. This could be explained by the fact that five of them belong to the same subgenus group: *Anoplophytum* (*T. aeranthos*, *T; bergeri*, *T. aeranthos x T. bergeri*, *T. aeranthos ‘uruguay’,* and *T. tenuifolia*).

The olfactory sensitivity and system are not the same between humans and pollinators. Moreover, between humans, the detection level of odors can vary. The combination of VOCs emitted by a plant allows the pollinators to extract information about the plant species, the nectar available, and its quality [33]. For instance, pollutants present or accumulated by plants may affect their emission of VOCs and how pollinators react to this phenomenon. The emission of VOCs can be different depending on the action the plant needs. Thus, VOCs could be emitted as inhibitors of bacterial growth or to reduce the threat of herbivores such as insects and larvae [34].

One of the functions of the emission of VOCs from a flower’s plant is the attraction of pollinators. Pollinators like hummingbirds, bats, moths, and bees are known in the *Tillandsia* genus [35,36,37]. Their attraction is most of the time very specific [11]. Consequently, emission of VOCs from scentless flowers as the ones studied here can have an ecological role like pollinators attractors, together with the shape and color of the scentless flowers. The eight major compounds identified in the *Tillandsia* species (except *T. paleacea* and *T. bandensis*) were, for most of them, not identified in our previous study conducted by Mame Lo et al. [26,27]. Only benzaldehyde (22) and benzacetaldehyde (24) were previously identified, meaning that these compounds might be specific to the scentless or faint-scented species of the *Tillandsia* genus or were not detected in our previous study because they were minor. This difference in the composition of volatile profiles could also be confirmed by the attraction of different pollinators [38,39,40]. Indeed, hummingbirds are more attracted by non-scented or faint-scented flowers [41].

Among the volatile compounds identified here, 8 of them are present in the majority of the 14 species and nonanal (49) was identified in all the species. In comparison, two of the eight compounds were identified in our previous studies (benzaldehyde (22) and benzacetaldehyde (24)). Nonanal (49) isolated from plants has already been studied from *Solanum lycopersicum* for its anti-fungal activity against *Penicilium cyclopium* and from *Artemisia ludoviciana* for anti-diarrheal activity [42,43]. Other aliphatic aldehydes were identified in this study such as hexanal (43), heptanal (44), octanal (47), decanal (51), 2-heptenal (45), 2-octenal (48), or 2-nonenal (50), for instance. Some of these aliphatic aldehydes were already studied for their in vitro antibacterial activity from *Olea europea L.* [44]. Last but not least, some of these aldehydes such as nonanal (49), octanal (47)—but also other compounds isolated in this study like 3-hexen-1-ol (39) and methyl salicylate (28)—were also studied for their plant–insect interaction and appeared to have a good attraction power for beneficial insects [45]. Usually, aldehydes are often known for their contribution to the aroma and taste of fruits or plants [46]. 2-pentylfuran (59) has also been identified in 10 of the 14 *Tillandsia* species studied here. This compound is described in the literature to be a repellent of *Drosophila suzukii* in order to avoid the use of insecticides [47]. Consequently, the emission of 2-pentylfuran (59) by 10 of the *Tillandsia* species studied here could be linked to a repellent activity. In the same way, benzaldehyde (24), a well-known volatile compound, was identified in this study and has been described for its herbicidal activity from *Melaleuca cajeputi* [48].

## 4. Materials and Methods

### 4.1. Plant Material and Chemicals

All the flowers of the *Tillandsia* species used for this study come and are available from the *Tillandsia* PROD plant nursery located in Le Cailar (Occitanie, France, 43°41′31.98″ N, 4°14′34.85″ E) and were harvested from April to September 2021. We used 14 species, hybrids and forms: *T. aeranthos*, *T. bergeri*, *T. aeranthos x T. bergeri* (hybrids of both species), *T. aeranthos ‘uruguay’* (a purple foliage form of *T. aeranthos*), *T. albertiana, T. ixioides, T. ionantha, T. tenuifolia, T. paleacea, T. cacticola, T. lorentziana, T. didisticha, T. bandensis*, and *T. recurvata*. For *T. tenuifolia*, 8 plants were used, providing 2 flowers for each analysis. For *T. albertiana, T. ixioides, T. paleacea, T. recurvata*, and *T. lorentziana*, 5 plants were used, providing 2 flowers each (except for *T. recurvata* which 3 flowers were provided). For *T. ionantha*, 12 plants were used, providing 2 flowers each. For *T. aeranthos*, *T. bergeri,* and *T. aeranthos x T. bergeri*, respectively 7, 18, and 17 plants were used, providing 2 flowers for each. For *T. cacticola*, 10 plants were used, providing 2 flowers for each. For *T. didisticha*, 6 plants were used with 2 flowers each. For *T. bandensis*, 9 plants were used, providing 3 flowers each. For *T. aeranthos ‘uruguay’*, 4 plants were used, providing 2 flowers each. The chemicals used for the identification of the compounds were ordered from Sigma-Aldrich (St. Louis, MO, USA): α-pinene (98.5% purity), β-pinene (98.5%), β-myrcene (90%), limonene (99%), eucalyptol (99%), β-ocimene (95.4%), γ-terpinene (98.5%), α-terpineol (90%), 1,4-cineole (98.5%), terpinolene (94%), α-ionone (96%), benzaldehyde (99.5%), phenylacetaldehyde (95%), methyl phenylacetate (97%), methyl benzoate (99.5%), benzyl acetate (99.7%), benzyl alcohol (99.9%), 2-phenylethanol (99%), 2-methoxy-4-vinylphenol (98%), methyl salicylate (99%), phenylethyl acetate (97%), eugenol (99.6%), methyl eugenol (98%), benzyl benzoate (98%), hexyl acetate (99.7%), methyl nicotinate (98%), isoamyl alcohol (99.8%), isoamyl acetate (99.7%), 2-pentylfuran (98%), indole (99.9%), nonanoic acid (99.5%), pentanol (99.8%), hexanol (99.9%), heptanol (99.9%), octanol (98%), hexanal (95%), heptanal (95%), 2-heptenal (95%), octanal (98%), 2-octenal (97%), nonanal (97%), 2-nonenal (95%), decanal (95%), and undecanal (98%).

### 4.2. HS-SPME Conditions

In previous studies, the Carboxen / Polydimethylsiloxane (CAR/PDMS) 75 µm fiber was determined to be the most appropriate for extracting the maximum number of volatile compounds emitted by *T. xiphioides, T. crocata*, and *T. caliginosa* flowers. In this study, different fibers have been tested on *T. bergeri* flowers. Between Carboxen/Polydimethylsiloxane (CAR/PDMS), Polydimethylsiloxane (PDMS) and Carboxen/Polydimethylsiloxane/Divinylbenzene (CAR/PDMS/DVB), respectively, 16, 3, and 12 compounds have been extracted with the second extraction method detailed below. Consequently, the CAR/PDMS fiber appeared to be the fiber that extracts the more volatile compound emitted by non-fragrant *T. bergeri* flowers, so this fiber was used for the present study of 14 species of *Tillandsia* flowers. The CAR/PDMS and the SPME device were ordered from Supelco (Bellefonte, PA, USA). Prior to use, the fiber was conditioned according to the manufacturer’s recommendations. Extractions were performed by placing a flower in a 20 mL amber vial sealed with a polytetrafluoroethylene (PTFE) septum-lined cap. For each *Tillandsia* flower, two successive extractions were performed: a first extraction method of 20 min at a temperature of 30 °C and a second extraction method of 65 min at 75 °C. These extraction methods provide a global view of the volatile compounds present in *Tillandsia* flowers and were retained after the optimization of the SPME method performed during our previous study. For both extraction methods, the equilibrium and desorption times are, respectively, 7.5 and 4 min.

### 4.3. Instrumentation and GC-MS Conditions

The analysis was performed on an Agilent 7890 B gas chromatograph coupled with an Agilent 5977 A mass spectrometer (Agilent Technologies, Santa Clara, CA, USA) with an MPS autosampler, a thermal desorption unit (TDU), and a cooled injection system (CIS) (Gerstel, Mülheim, Germany). MSD Chemstation version F.01.00 data acquisition software (Agilent Technologies, Santa Clara, CA, USA) was used to program the GC-MS. After extraction, desorption was performed thermally in the TDU at the recommended temperature of 300 °C for CAR/PDMS. After desorption, the injection was performed in split mode; the analytes were focused on the CIS at −10 °C for 2 min, then brought to 250 °C at a heating rate of 12 °C per second and maintained for 2.5 min. A DB-5MS capillary column (5% diphenyl cross-linked 95% dimethylpolysiloxane, 30 m × 0.25 mm × 0.25 µm) (Agilent Technologies, Santa Clara, CA, USA) was used and the separation conditions were as follows: initial column temperature of 40 °C for 2 min, then increased by 4 °C/min to 130 °C for 1 min, then increased by 7 °C/min to 230 °C, where it was maintained for 4 min. High purity Helium (99.999%) was used as the carrier gas at a flow rate of 1.2 mL per minute. The temperature of the transfer line was set at 250 °C and the temperature of the ion source at 230 °C. The ions were generated by a 70 eV electron beam. The mass range was scanned from m/z 33 to 500 Da.

### 4.4. Identification

MassHunter Qualitative Analysis version B.06.00 (Agilent Technologies, Santa Clara, CA, USA) was used to integrate the peaks of the chromatograms being above the analytical noise. First, the volatile compounds were identified by comparing their mass spectra to those of compounds in the commercial databases, National Institute of Standard and Technologies (NIST) and Wiley11 (R > 800). Then, the retention indices of the compounds were calculated relative to the n-alkanes (C7–C30) and compared to those of compounds in the NIST online database (https://webbook.nisT.gov/chemistry/cas-ser.html)(accessed on 28 August 2021). Finally, the identifications were confirmed by injecting the available standards (mentioned above) into the GC-MS based on the comparison of mass spectra and retention times. For the standard solutions, 0.2 µL of each standard was added in 2 mL of hexane, and the solvent was subsequently evaporated with nitrogen. The analysis was performed under the same conditions as for the flowers.

### 4.5. Statistical Analysis

For statistical analysis, peak area values of the total ion chromatograms were measured with MassHunter and transferred to Excel (Microsoft Excel 2013, version 15.0.5363.1000). For this purpose, for each plant providing two flowers, an average of the areas of the peaks of the compounds was calculated in Excel. Principal component analysis (PCA) using FactoMiner pakage (version 2.4) and heatmap using the pheatmap package (version 1.0.12) were performed with the R software (version 3.6.3, company Foundation for Statistical Computing, Vienna, Austria) based on a univariate scaling method. Heatmap data was clustered using Ward’s method. Odor characteristics were obtained from the “The Good Scents” company network database (www.thegoodscentscompany.com, accessed on 27 August 2021).

## 5. Conclusions

This study allowed the identification of 65 volatile organic compounds (monoterpenes, sesquiterpenes, phenylpropanoids/benzenoids, alcohols, aldehydes, and other compounds) in 14 scentless or faint-scented species of *Tillandsia* with only 21 pooled compounds with our previous study. The extraction and identification of these compounds were performed using a HS-SPME/GC-MS system using two different extraction methods developed in a previous study in our laboratory. Six to 25 compounds were identified depending on the species. A qualitative heatmap and two PCA were used to compare the profiles of the 14 species studied. Some of the identified compounds were only identified in non-fragrant *Tillandsia* species such as aliphatic aldehydes and alcohols, which shows an originality in the compounds identified compared to our previous study about the fragrant *Tillandsia* species. The comparison of the profiles showed differences between some species. For instance, faint-scented *T. paleacea* and *T. bandensis* have a different profile between each other and also compared to the 12 other species. Concerning the other species, the profile showed few differences such as sesquiterpenoids present in *T. aeranthos* and phenylpropanoids/benzenoids in *T. bergeri,* for example, although the profiles are quite similar. Some of the VOCs identified have been described in the literature for their activities as repellent or attractor of pollinators. This may lead to conclude that even non-fragrant flowers and plants for human beings have an important ecological role and also a potential therapeutic role thanks to the VOCs emitted. Our studies show the large diversity of VOCs emitted by the flowers in the *Tillandsia* genus and could be of help in the comprehension of the ecological role and function of these volatile compounds in the interactions of these plants with their environment.

## Figures and Tables

**Table 1:** Flowers of the 14 Tillandsia species studied. © Julien Vigo for the photographs.

Name	*T.aeranthos*	*T. bergeri*	*T. aeranthos* x *bergeri*	*T. aeranthos ‘uruguay’*	*T. albertiana*
	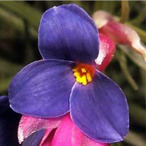	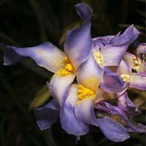	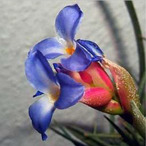	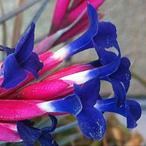	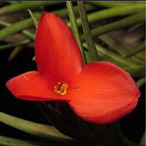
**Name**	***T. ixioides***	***T. ionantha***	***T. tenuifolia***	***T. paleacea***	***T. cacticola***
	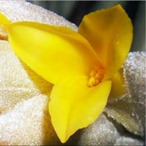	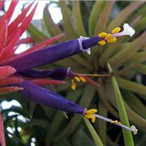	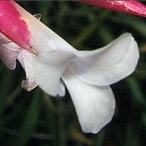	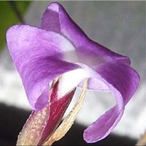	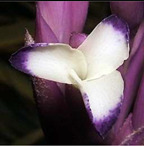
**Name**	***T. lorentziana***	***T. didisticha***	***T. bandensis***	***T. recurvata***	
	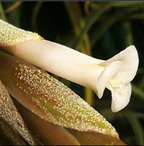	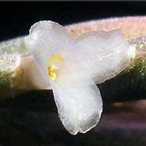	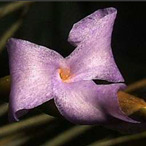	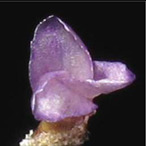	

**Table 2:** Identification of the volatile organic compounds from floral emissions of the 14 Tillandsia species studied.

					Area (× 10^3^)													
#	Family	Compounds	RT (min)	RI	*Aeranthos*	*Bergeri*	*Aeranthos x bergeri*	*Aeranthos ‘uruguay’*	*Albertiana*	*Ixioides*	*Ionantha*	*Tenuifolia*	*Paleacea*	*Cacticola*	*Lorentziana*	*Didisticha*	*Bandensis*	*Recurvata*
1	M	cosmene ^a,c^	17.26	968	nd	nd	nd	nd	nd	32.34	nd	nd	nd	nd	nd	nd	nd	nd
2	M	β-myrcene ^a,e^	17.64	978	nd	nd	nd	nd	nd	nd	nd	nd	8.76	nd	nd	nd	nd	nd
3	M	β-pinene ^a,e^	17.68	980	nd	nd	nd	nd	nd	nd	nd	nd	27.42	nd	nd	nd	nd	35.20
4	M	1,4-cineole ^a,f^	17.77	982	nd	110.47	nd	nd	nd	nd	nd	nd	nd	nd	nd	nd	nd	nd
5	M	*o*-cymene ^a,d^	18.67	1006	nd	nd	nd	nd	nd	44.35	nd	nd	60.47	nd	nd	nd	nd	nd
6	M	eucalyptol ^a,e^	19.43	1026	nd	nd	nd	nd	nd	nd	nd	nd	1774.46	nd	nd	nd	nd	nd
7	M	isomyocorene ^a,c^	19.09	1017	nd	nd	nd	nd	nd	nd	nd	nd	nd	nd	nd	nd	165.23	nd
8	M	limonene ^a,e^	19.27	1022	nd	nd	nd	nd	nd	nd	nd	nd	192.85	nd	nd	nd	nd	nd
9	M	β-ocimene ^a,f^	19.75	1035	nd	nd	nd	nd	nd	464.37	nd	nd	153.83	nd	nd	nd	4637.66	nd
10	M	γ-terpinene ^a,e^	20.57	1056	nd	nd	nd	nd	nd	nd	nd	nd	nd	nd	nd	nd	174.52	nd
11	M	terpinolene ^a,e^	21.48	1081	nd	nd	nd	nd	nd	nd	nd	nd	nd	nd	nd	nd	138.67	nd
12	M	α-terpineol ^b,e^	26.07	1204	nd	nd	nd	nd	nd	nd	nd	nd	87.03	nd	nd	nd	nd	nd
13	S	α-copaene ^b,d^	31.18	1374	90.65	nd	nd	nd	nd	nd	nd	nd	nd	824.63	nd	nd	nd	nd
14	S	δ-selinene ^a,c^	31.31	1379	nd	nd	nd	22.09	nd	nd	nd	nd	nd	nd	nd	nd	nd	nd
15	S	α-guaiene ^b,d^	32.00	1406	nd	nd	nd	nd	1067.07	nd	nd	nd	nd	nd	nd	nd	nd	nd
16	S	rotundene ^b,c^	32.71	1439	424.64	nd	nd	671.00	nd	nd	nd	nd	nd	nd	nd	nd	nd	nd
17	S	caryophyllene ^b,d^	32.91	1448	nd	nd	nd	nd	nd	nd	nd	nd	nd	1413.36	nd	nd	nd	nd
18	S	humulene ^b,d^	33.51	1475	nd	nd	nd	nd	nd	nd	nd	nd	nd	728.64	nd	nd	nd	nd
19	S	α-muurolene ^b,d^	33.69	1484	nd	nd	nd	226.09	nd	nd	nd	nd	nd	nd	nd	nd	nd	nd
20	S	trans-β-bergamotene ^b,d^	32.72	1439	nd	nd	nd	nd	nd	nd	nd	nd	nd	nd	nd	nd	nd	58.06
21	S	trans-calamenene ^b,d^	33.91	1494	38.73	nd	nd	nd	nd	nd	nd	nd	nd	nd	nd	nd	nd	nd
22	P/B	benzaldehyde ^b,e^	16.33	943	133.82	4031.24	385.83	193.67	400.61	1132.68	nd	nd	81.53	326.21	137.91	160.00	299.20	2249.88
23	P/B	benzyl alcohol ^b,e^	19.09	1017	nd	13875.31	1743.88	nd	nd	nd	nd	nd	nd	1163.51	84.46	nd	nd	7269.54
24	P/B	benzacetaldehyde ^b,e^	19.57	1029	193.65	nd	nd	258.54	620.21	9030.12	184.75	274.36	224.50	nd	220.17	342.89	nd	210.03
25	P/B	methyl benzoate ^a,e^	20.70	1060	nd	18082.32	3736.94	37.51	nd	nd	nd	nd	nd	nd	nd	nd	nd	nd
26	P/B	2-phenylethanol ^b,e^	21.72	1087	207.02	317.23	93.45	nd	nd	nd	nd	nd	92.60	nd	nd	222.72	nd	437.85
27	P/B	benzyl acetate ^b,e^	23.37	1131	nd	123.90	52.76	nd	nd	nd	nd	nd	52.05	nd	nd	nd	nd	4502.82
28	P/B	methyl salicylate ^b,e^	25.75	1195	nd	nd	nd	nd	nd	nd	nd	nd	nd	807.36	nd	nd	nd	nd
29	P/B	phenylethyl acetate ^b,e^	27.64	1252	nd	nd	nd	nd	nd	nd	nd	nd	155.02	nd	nd	nd	nd	778.30
30	P/B	Eugenol ^b,e^	29.70	1318	nd	nd	nd	nd	nd	nd	nd	nd	nd	nd	nd	nd	50.60	nd
31	P/B	2-methoxy-4-vinylphenol ^b,e^	29.74	1316	nd	nd	nd	nd	nd	40.77	nd	nd	nd	nd	nd	98.30	nd	nd
32	P/B	methyl anthranilate ^b,d^	29.94	1327	nd	85.02	nd	nd	nd	nd	nd	nd	nd	nd	nd	nd	52.00	nd
33	P/B	benzyl butyrate ^b,c^	29.99	1329	nd	nd	nd	nd	nd	nd	nd	nd	23.80	nd	nd	nd	nd	nd
34	P/B	butyl benzoate ^b,c^	30.80	1360	nd	nd	nd	nd	nd	nd	nd	nd	111.80	nd	nd	nd	nd	nd
35	P/B	methyl ortho-anisate ^b,c^	31.16	1368	nd	nd	nd	nd	nd	nd	nd	nd	nd	nd	nd	nd	5918.75	nd
36	P/B	benzyl isovalerate ^b,c^	31.31	1375	nd	nd	nd	nd	nd	nd	nd	nd	96.73	nd	nd	nd	nd	nd
37	P/B	methyl eugenol ^b,e^	31.77	1397	nd	nd	nd	nd	nd	nd	nd	nd	nd	nd	nd	nd	55.32	nd
38	Al	pentanol ^b,f^	9.34	-	105.89	nd	nd	354.79	nd	nd	nd	nd	nd	nd	nd	nd	nd	nd
39	Al	3-hexen-1-ol ^b,c^	12.42	839	nd	nd	nd	nd	nd	nd	nd	nd	nd	nd	nd	nd	nd	173.37
40	Al	hexanol ^b,e^	12.74	847	45.21	326.40	141.18	401.11	1138.06	312.20	524.56	140.16	75.12	306.41	26.78	nd	nd	nd
41	Al	octanol ^b,e^	20.80	1063	nd	nd	73.07	nd	nd	nd	871.99	175.51	43.54	234.54	107.55	26.53	nd	nd
42	Al	hexadecanol ^b,c^	37.26	1676	nd	nd	nd	nd	nd	nd	nd	nd	nd	nd	nd	nd	92.36	nd
43	A	hexanal ^b,e^	10.15	-	832.91	394.28	354.29	1906.08	3379.54	1969.27	1445.37	326.61	158.25	456.87	373.34	nd	nd	nd
44	A	heptanal ^b,e^	14.27	888	98.78	657.01	415.61	2468.93	6175.15	1620.57	2714.32	415.83	103.58	996.29	89.93	nd	nd	110.01
45	A	2-heptenal ^b,e^	15.95	933	41.32	nd	25.90	66.35	124.46	169.04	43.41	nd	nd	nd	nd	nd	nd	nd
46	A	2,4-heptadienal ^b,d^	17.36	971	nd	nd	nd	nd	nd	46.91	nd	nd	nd	nd	nd	nd	nd	nd
47	A	octanal ^b,e^	17.95	987	10.21	nd	189.91	812.11	734.32	143.54	1396.31	77.24	73.62	239.37	150.42	nd	nd	nd
48	A	2-octenal ^b,e^	20.40	1052	nd	nd	26.05	136.31	180.46	162.36	118.13	nd	nd	nd	nd	nd	nd	nd
49	A	nonanal ^b,e^	21.91	1092	40.12	240.81	351.16	2145.51	2451.38	1352.68	4072.88	484.48	266.99	1007.57	462.02	208.01	47.73	201.69
50	A	2-nonenal ^b,f^	23.62	1138	nd	nd	20.61	105.50	181.02	153.78	88.97	nd	5.25	64.20	27.86	nd	nd	nd
51	A	decanal ^b,e^	25.58	1191	111.81	nd	19.33	76.57	422.97	140.92	88.81	nd	16.56	42.47	45.78	nd	nd	nd
52	A	2-decenal ^b,c^	26.93	1231	nd	nd	nd	nd	99.98	nd	nd	nd	nd	nd	nd	nd	nd	nd
53	A	undecanal ^b,e^	28.23	1270	nd	nd	nd	nd	62.21	26.70	nd	nd	nd	nd	nd	nd	nd	nd
54	A	tetradecanal ^b,d^	36.17	1612	nd	nd	nd	nd	nd	nd	nd	nd	nd	nd	nd	nd	184.12	nd
55	A	hexadecanal ^b,d^	39.51	1819	nd	nd	nd	nd	nd	nd	nd	nd	nd	nd	nd	nd	478.86	nd
56	O	isoamyl acetate ^a,e^	13.15	858	nd	nd	nd	nd	nd	107.82	nd	nd	nd	nd	nd	nd	nd	78.84
57	O	methional ^b,d^	14.24	887	nd	nd	nd	nd	nd	2795.63	nd	nd	nd	nd	nd	nd	nd	40.75
58	O	sulcatone ^b,c^	16.45	947	nd	nd	nd	nd	228.81	284.38	nd	nd	nd	nd	nd	nd	nd	nd
59	O	furan-2-pentyl ^b,e^	17.20	967	185.82	219.40	193.96	230.93	151.65	482.68	133.69	36.03	32.06	nd	86.61	nd	nd	nd
60	O	hexyl acetate ^a,e^	18.43	1000	nd	nd	nd	nd	nd	nd	nd	nd	14.96	nd	nd	nd	nd	188.81
61	O	methyl nicotinate ^b,f^	23.62	1138	nd	nd	nd	nd	nd	nd	nd	nd	nd	nd	nd	nd	253.44	nd
62	O	nonanoic acid ^b,e^	26.64	1222	nd	nd	nd	nd	nd	162.19	nd	nd	nd	nd	nd	nd	nd	nd
63	O	Indole ^b,e^	27.92	1261	nd	nd	404.59	nd	nd	nd	nd	nd	nd	nd	nd	nd	1393.13	1113.55
64	O	α-ionone ^b,e^	31.45	1384	nd	nd	76.52	nd	101.45	nd	nd	nd	nd	nd	nd	nd	nd	nd
65	O	trans-β-ionone ^b,d^	32.76	1441	nd	nd	nd	nd	551.77	108.04	nd	nd	nd	nd	nd	nd	nd	nd
		Number of detected compounds per species			15	12	18	17	18	23	12	8	25	14	12	6	15	15

# = compound number, nd = non detected, M = Monoterpene, S = Sesquiterpene, P/B = Phenylpropanoid/Benzenoids, Al = Alcohol, A = Aldehyde, O = Other, a: efficient extraction with the first method (30 °C and 20 min), b: efficient extraction with the second method (75 °C and 65 min), c: identification performed by comparing the mass spectrum with that of the NIST library, d: identification performed by comparing the mass spectrum with that of the NIST library and by comparison of RI (retention index) with RI of published literatures and online library (https://webbook.nist.gov/chemistry/cas-ser.html, accessed on 28 August 2021), e: identification performed by comparing the mass spectrum with that of the NIST library, by comparison of RI (retention index) with RI of published literatures and online library and by comparison of the retention time and mass spectrum of the authentic standard, f: identification performed by comparing the mass spectrum with that of the NIST library and by comparison of the retention time and mass spectrum of the authentic standard. For the complete table, see Table S1 in the Supplementary Materials.

## Data Availability

The data presented in this study are available within the article and Supplementary Materials.

## Supplementary Materials

The following are available online at https://www.mdpi.com/article/10.3390/metabo12070628/s1, Figure S1: Chromatograms obtained using the first extraction method; Figure S2: Chromatograms obtained using the second extraction method, Table S1: Complete table of the identification of volatile compounds from floral emissions of the 14 Tillandsia species.
