# Supplementary material for: Extraction and Identification of Volatile Organic Compounds in Scentless Flowers of 14 Tillandsia Species Using HS-SPME/GC-MS
Source: Metabolites. 2022 Jul 8;12(7):628. doi: 10.3390/metabo12070628 (PMC9316202; doi:10.3390/metabo12070628)
Supplement: Supplementary file 1 [file metabolites-12-00628-s001.zip › metabolites-1763633-supplementary.pdf]

Supplementary Material

Extraction and Identification of Volatile Organic Compounds in Scentless Flowers of Fourteen *Tillandsia* Species Using HS-SPME/GC-MS

Alexandre Gonzalez [1], Zohra Benfodda [1], David Bénimélis [1], Jean-Xavier Fontaine [2], Roland Molinié [2] and Patrick Meffre [1,*]

[1] UNIV. NIMES, UPR CHROME, CEDEX 1, F-30021 Nîmes, France; alexandre.gonzalez@unimes.fr (A.G.); zohra.benfodda@unimes.fr (Z.B.); david.benimelis@unimes.fr (D.B.)
[2] UMR INRAE 1158 Transfrontalière BioEcoAgro, BIOlogie des Plantes et Innovation (BIOPI), UPJV, UFR de Pharmacie, 80037 Amiens, France; jean-xavier.fontaine@u-picardie.fr (J.-X.F.); roland.molinie@u-picardie.fr (R.M.)
* Correspondence: patrick.meffre@unimes.fr

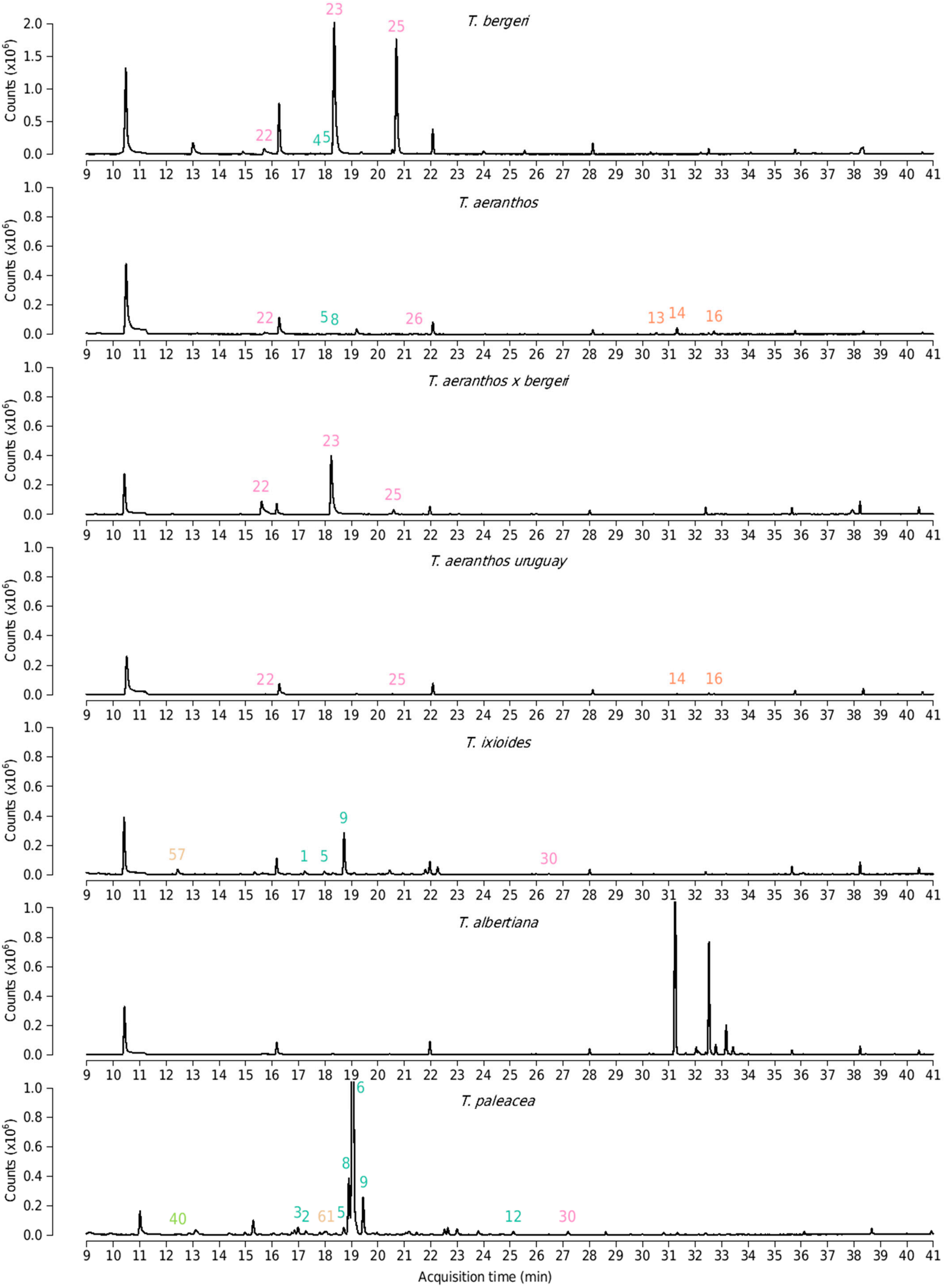
T. bergeri
T. aeranthos
T. aeranthos x bergeri
T. aeranthos uruguay
T. ixioides
T. albertiana
T. paleacea
Counts (x10^6)
Acquisition time (min)
23
25
22
4
5
8
26
13
14
16
57
1
9
30
6
40
3
2
61
12

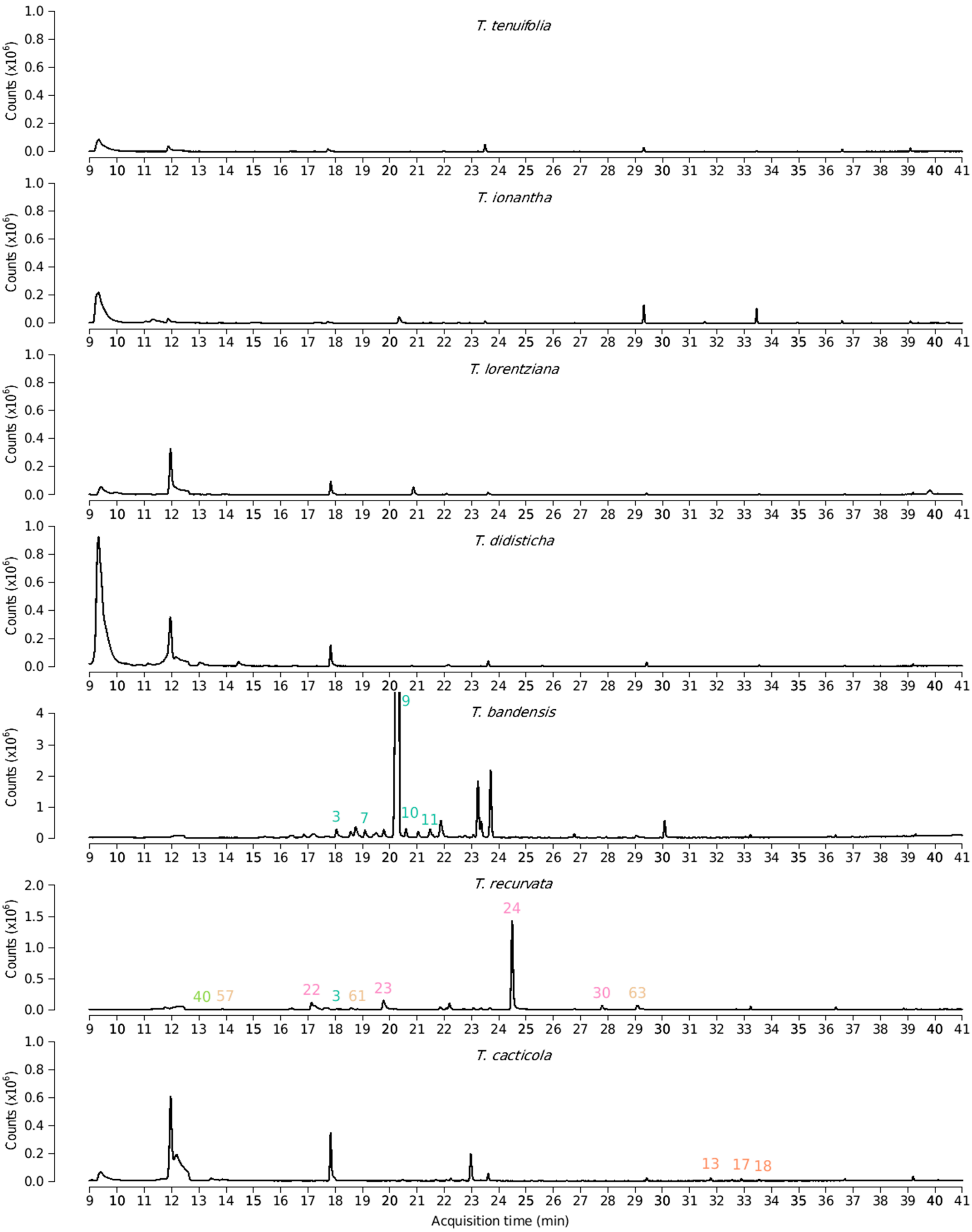

Figure S1: Chromatograms obtained using the first extraction method (low values of temperature and extraction time). Chromatographic separation was carried out on a DB-5MS column and the extraction were performed with CAR/PDMS fiber for 20 minutes at an extraction temperature of 30 °C. The identification numbers correspond to

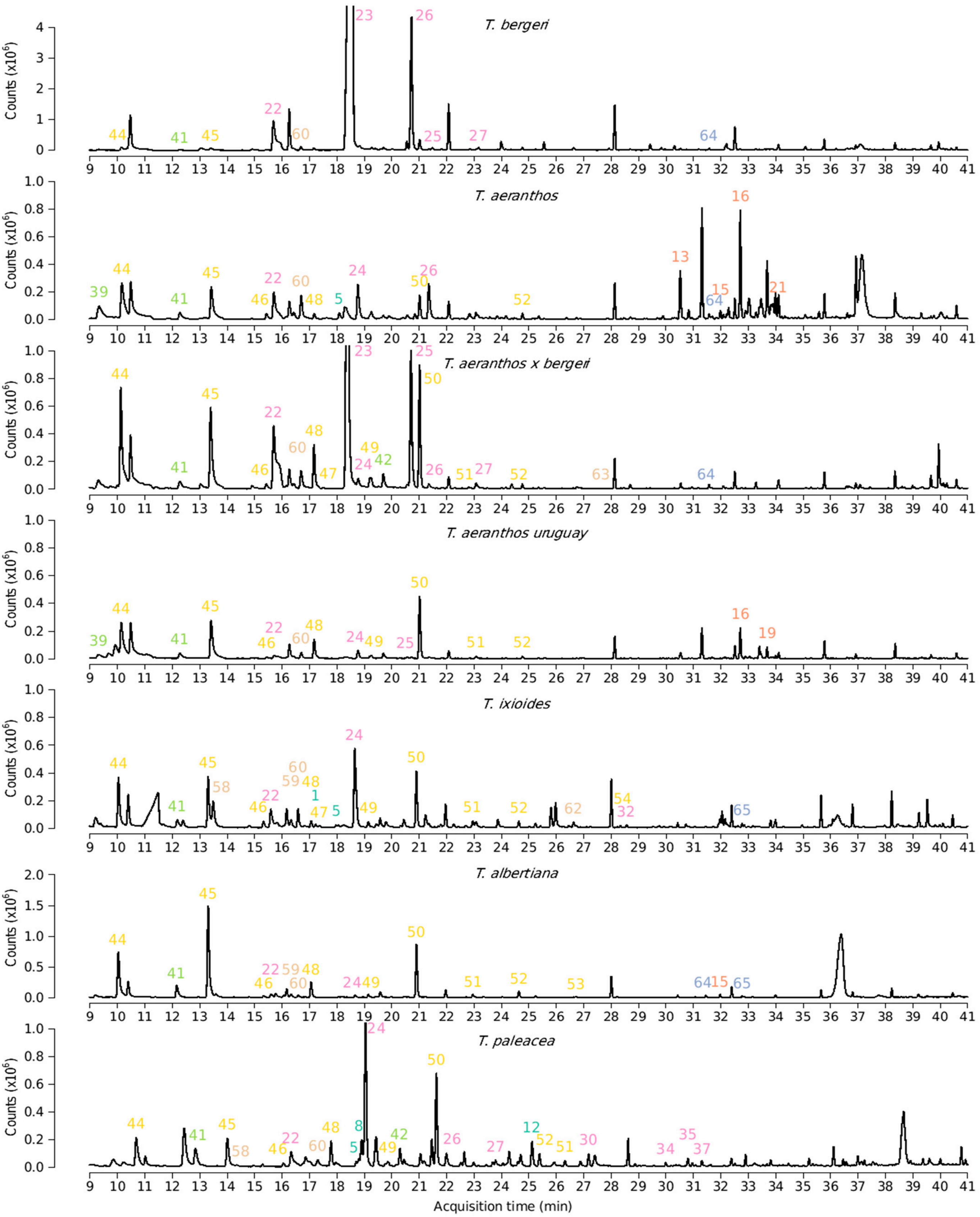
T. bergeri
44 41 45 22 60 23 26 25 27 64
T. aeranthos
39 44 41 45 46 22 60 48 5 24 50 26 52 13 64 15 16 21
T. aeranthos x bergeri
44 41 45 46 22 60 48 47 24 49 42 23 25 50 26 51 27 52 63 64
T. aeranthos uruguay
39 44 41 45 46 22 60 48 24 49 25 50 51 52 16 19
T. ixioides
44 41 45 58 46 22 59 60 48 1 47 5 24 49 50 51 52 62 54 32 65
T. albertiana
44 41 45 46 22 59 60 48 24 49 50 51 52 53 64 15 65
T. paleacea
44 41 45 58 46 22 60 48 5 8 24 49 42 50 26 27 12 52 51 30 34 35 37
Counts (x10^6)
Acquisition time (min)

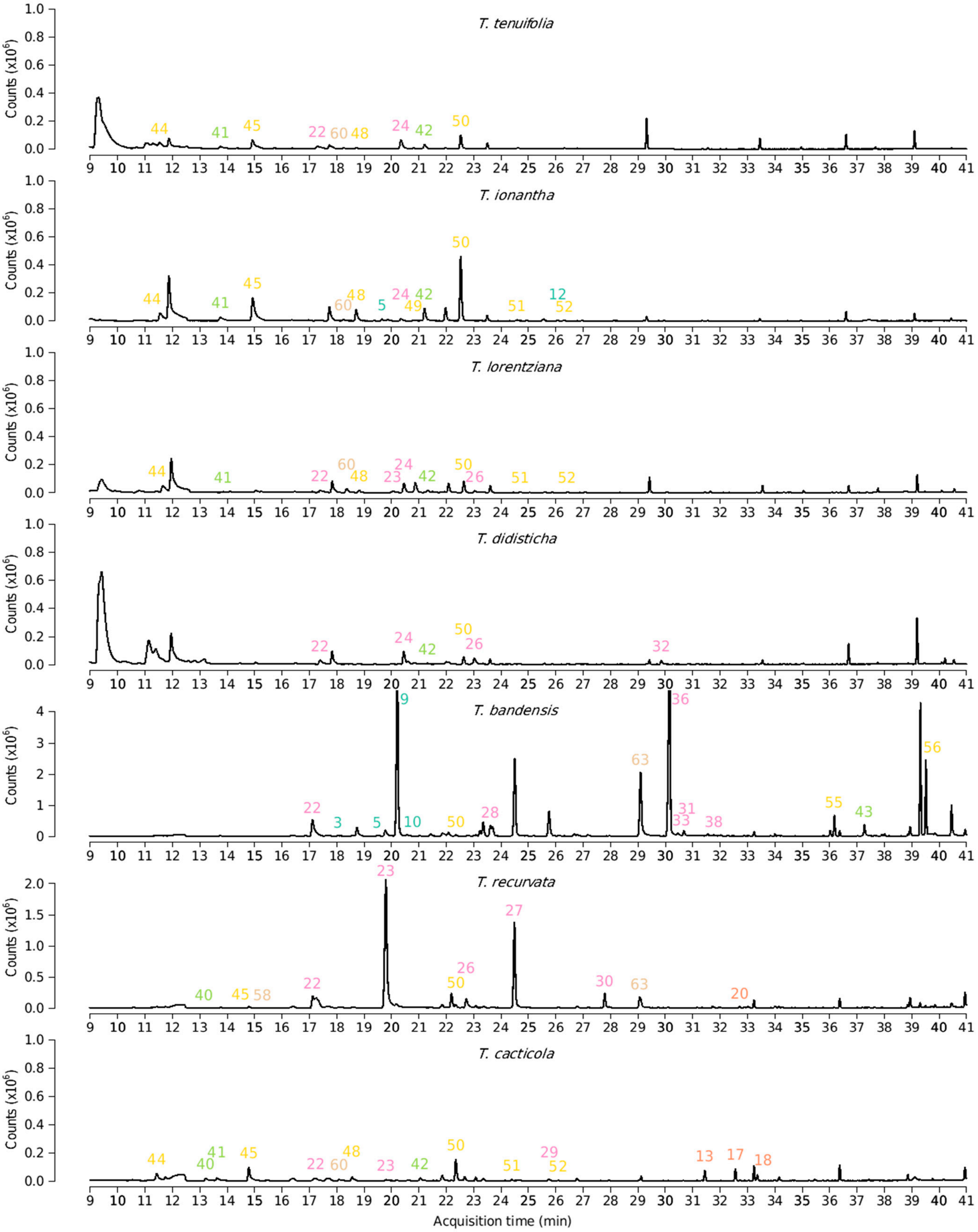

Figure S2: Chromatograms obtained using the second extraction method (high values of temperature and extraction time). Chromatographic separation was carried out on a DB-5MS column and the extraction were performed with CAR/PDMS fiber for 65 minutes at an extraction temperature of 75 °C. The identification numbers correspond to those shown in **Table 2** and in **Table S1**.

Table S1: Complete table of the identification of volatile compounds from floral emissions of the fourteen *Tillandsia* species.

						Area ± SD (x 10^3)													
#	Family	Compounds	RT (min)	RI	Odor[g]	*aeranthos*	*bergeri*	*aeranthos x bergeri*	*aeranthos uruguay*	*albertiana*	*ixiooides*	*ionantha*	*tenuifolia*	*paleacea*	*cacticola*	*lorentziana*	*didisticha*	*bandensis*	*recurvata*
1	M	cosmene[a,c]	17.26	968	-	nd	nd	nd	nd	nd	32.34 ±23.5	nd	nd	nd	nd	nd	nd	nd	nd
2	M	β-myrcene[a,e]	17.64	978	pepper, spice, balsamic	nd	nd	nd	nd	nd	nd	nd	nd	8.76 ±7.8	nd	nd	nd	nd	nd
3	M	β-pinene[a,e]	17.68	980	dry, pine, woody	nd	nd	nd	nd	nd	nd	nd	nd	27.42 ±16.1	nd	nd	nd	nd	35.20 ±13.7
4	M	1,4-cineole[a,f]	17.77	982	pine, mint, herb	nd	110.47 ±115.9	nd	nd	nd	nd	nd	nd	nd	nd	nd	nd	nd	nd
5	M	o-cymene[a,d]	18.67	1006	-	nd	nd	nd	nd	nd	44.35 ±49.2	nd	nd	60.47 ±26.8	nd	nd	nd	nd	nd
6	M	eucalyptol[a,e]	19.43	1026	herbal	nd	nd	nd	nd	nd	nd	nd	nd	1774.46 ±730.5	nd	nd	nd	nd	nd
7	M	isomyocorene[a,c]	19.09	1017	-	nd	nd	nd	nd	nd	nd	nd	nd	nd	nd	nd	nd	165.23 ±90.3	nd
8	M	limonene[a,e]	19.27	1022	citrus, orange, fresh sweet	nd	nd	nd	nd	nd	nd	nd	nd	192.85±121.4	nd	nd	nd	nd	nd
9	M	β-ocimene[a,f]	19.75	1035	citrus, tropical herb, woody	nd	nd	nd	nd	nd	464.37 ±479.3	nd	nd	153.83 ±109.1	nd	nd	nd	4637.66 ±2570.8	nd
10	M	γ-terpinene[a,e]	20.57	1056	oil, woody, lemon, tropical herb	nd	nd	nd	nd	nd	nd	nd	nd	nd	nd	nd	nd	174.52±91.5	nd
11	M	terpinolene[a,e]	21.48	1081	wood, sweet pine, citrus	nd	nd	nd	nd	nd	nd	nd	nd	nd	nd	nd	nd	138.67 ±61.3	nd
12	M	α-terpineol[b,e]	26.07	1204	pine, turpentine, citrus, floral, wood	nd	nd	nd	nd	nd	nd	nd	nd	87.03 ±91.1	nd	nd	nd	nd	nd

13	S	α-copaene[b,d]	31.18	1374	woody, spice, honey	90.65 ±34.2	nd	nd	nd	nd	nd	nd	nd	nd	824.63 ±681.4	nd	nd	nd	nd
14	S	δ-selinene[a,c]	31.31	1379	-	nd	nd	nd	22.09 ±22.7	nd	nd	nd	nd	nd	nd	nd	nd	nd	nd
15	S	α-guaiene[b,d]	32.00	1406	sweet woody, balsam, pepper	nd	nd	nd	nd	1067.07 ±1210.7	nd	nd	nd	nd	nd	nd	nd	nd	nd
16	S	rotundene[b,c]	32.71	1439	-	424.64 ±124.0	nd	nd	671.00 ±241.1	nd	nd	nd	nd	nd	nd	nd	nd	nd	nd
17	S	caryophyllene[b,d]	32.91	1448	clove, dry, spice, sweet woody	nd	nd	nd	nd	nd	nd	nd	nd	nd	1413.36 ±1305.9	nd	nd	nd	nd
18	S	humulene[b,d]	33.51	1475	woody	nd	nd	nd	nd	nd	nd	nd	nd	nd	728.64 ±720.2	nd	nd	nd	nd
19	S	α-muurolene[b,d]	33.69	1484	-	nd	nd	nd	226.09 ±83.2	nd	nd	nd	nd	nd	nd	nd	nd	nd	nd
20	S	trans-β-bergamotene[b,d]	32.72	1439	-	nd	nd	nd	nd	nd	nd	nd	nd	nd	nd	nd	nd	nd	58.06 ±21.8
21	S	trans-calamenene[b,d]	33.91	1494	-	38.73 ±23.9	nd	nd	nd	nd	nd	nd	nd	nd	nd	nd	nd	nd	nd
22	P/B	benzaldehyde[b,e]	31.45	1384	sweet woody, floral, violet, tropical fruit	nd	nd	76.52 ±85.3	nd	101.45 ±3.7	nd	nd	nd	nd	nd	nd	nd	nd	nd
23	P/B	benzyl alcohol[b,e]	32.76	1441	dry, floral, woody	nd	nd	nd	nd	551.77 ±502.6	108.04 ±25.5	nd	nd	nd	nd	nd	nd	nd	nd
24	P/B	benzacetaldehyde[b,e]	16.33	943	almond, cherry, sweet bitter, strong sharp	133.82 ±104.4	4031.24 ±3071.8	385.83 ±341.3	193.67 ±82.3	400.61 ±116.1	1132.68 ±305.6	nd	nd	81.53 ±85.1	326.21 ±431.8	137.91 ±92.0	160.00 ±60.4	299.20 ±314.3	2249.88 ±1007.1
25	P/B	methyl benzoate[a,e]	19.09	1017	floral, rose, balsamic	nd	13875.31 ±10151.2	1743.88 ±1846.6	nd	nd	nd	nd	nd	nd	1163.51 ±1249.9	84.46 ±101.1	nd	nd	7269.54 ±3938.0
26	P/B	2-phenylethanol[b,e]	19.57	1029	green, sweet floral, hyacinth, clover, honey, cocoa	193.65 ±208.2	nd	nd	258.54 ±53.7	620.21 ±395.9	9030.12 ±12797.4	184.75 ±153.7	274.36 ±170.6	224.50 ±72.8	nd	220.17 ±146.7	342.89 ±153.7	nd	210.03 ±53.8

27	P/B	benzyl acetate[b,e]	20.70	1060	cananga, almond, floral	nd	18082.32 ±14784.4	3736.94 ±6743.9	37.51 ±11.2	nd	nd	nd	nd	nd	nd	nd	nd	nd	nd
28	P/B	methyl salicylate[b,e]	23.37	1131	sweet floral, fruit, jasmin	nd	123.90 ±189.0	52.76 ±32.8	nd	nd	nd	nd	nd	52.05 ±76.8	nd	nd	nd	nd	4502.82 ±2595.1
29	P/B	phenylethyl acetate[b,e]	23.62	1138	warm, herbal, tobacco	nd	nd	nd	nd	nd	nd	nd	nd	nd	nd	nd	nd	253.44 ±137.1	nd
30	P/B	eugenol[b,e]	25.75	1195	green mint	nd	nd	nd	nd	nd	nd	nd	nd	nd	807.36 ±780.0	nd	nd	nd	nd
31	P/B	2-methoxy-4-vinylphenol[b,e]	27.64	1252	floral rose, sweet honey, tropical fruit	nd	nd	nd	nd	nd	nd	nd	nd	155.02 ±168.2	nd	nd	nd	nd	778.30 ±399.8
32	P/B	methyl anthranilate[b,d]	29.74	1316	dry, woody, fresh amber, cedar, roasted peanut	nd	nd	nd	nd	nd	40.77 ±5.8	nd	nd	nd	nd	nd	98.30 ±112.7	nd	nd
33	P/B	benzyl butyrate[b,c]	29.94	1327	fruit grape, orange flower, neroli	nd	85.02 ±43.2	nd	nd	nd	nd	nd	nd	nd	nd	nd	nd	52.00 ±45.1	nd
34	P/B	butyl benzoate[b,c]	29.99	1329	fresh, fruit, jasmin, apricot, loganberry	nd	nd	nd	nd	nd	nd	nd	nd	23.80 ±32.2	nd	nd	nd	nd	nd
35	P/B	methyl ortho-anisate[b,c]	30.80	1360	mild, amber, balsam, fruity	nd	nd	nd	nd	nd	nd	nd	nd	111.80 ±87.7	nd	nd	nd	nd	nd
36	P/B	benzyl isovalerate[b,c]	31.16	1368	herbal, floral, hyacinth, cananga, fruit, blackcurrant	nd	nd	nd	nd	nd	nd	nd	nd	nd	nd	nd	nd	5918.75 ±2855.1	nd
37	P/B	methyl eugenol[b,e]	31.31	1375	sweet, fruity, apple, pineapple, herbal	nd	nd	nd	nd	nd	nd	nd	nd	96.73 ±100.9	nd	nd	nd	nd	nd
38	Al	pentanol[b,f]	9.34	-	fusel, oil, sweet balsam	105.89 ±72.0	nd	nd	354.79 ±33.9	nd	nd	nd	nd	nd	nd	nd	nd	nd	nd

39	Al	3-hexen-1ol[b,c]	12.42	839	fresh green, cut grass, vegetable, herbal, oily	nd	nd	nd	nd	nd	nd	nd	nd	nd	nd	nd	nd	nd	173.37 ±132.3
40	Al	hexanol[b,e]	12.74	847	ethereal, fusel, oil, fruity alcoholic, sweet green	45.21 ±35.1	326.40 ±429.8	141.18 ±80.2	401.11 ±90.0	1138.06 ±666.6	312.20 ±97.9	524.56 ±442.6	140.16 ±82.8	75.12 ±65.2	306.41 ±98.6	26.78 ±32.5	nd	nd	nd
41	Al	octanol[b,e]	20.80	1063	waxy, green, orange, rose, mushroom	nd	nd	73.07 ±70.4	nd	nd	nd	871.99 ±695.5	175.51 ±126.1	43.54 ±35.3	234.54 ±67.3	107.55 ±47.9	26.53 ±3.6	nd	nd
42	Al	hexadecanol[b,c]	37.26	1676	wax, clean, greasy, floral, oil	nd	nd	nd	nd	nd	nd	nd	nd	nd	nd	nd	nd	92.36 ±42.0	nd
43	A	hexanal[b,e]	10.15	-	fresh green, grass, leaf, fruit sweaty	832.91 ±763.9	394.28 ±460.1	354.29 ±238.0	1906.08 ±304.1	3379.54 ±1345.0	1969.27 ±370.5	1445.37 ±1068.1	326.61 ±143.6	158.25 ±88.2	456.87 ±138.7	373.34 ±114.5	nd	nd	nd
44	A	heptanal[b,e]	14.27	888	green, herbal, wine-lee	98.78 ±88.7	657.01 ±851.0	415.61 ±150.1	2468.93 ±355.7	6175.15 ±3111.0	1620.57 ±470.5	2714.32 ±2600.0	415.83 ±201.7	103.58 ±69.8	996.29 ±259.3	89.93 ±115.2	nd	nd	110.01 ±33.5
45	A	2-heptenal[b,e]	15.95	933	pungent, green, vegetable, fresh	41.32 ±25.9	nd	25.90 ±11.1	66.35 ±12.3	124.46 ±58.3	169.04 ±46.5	43.41 ±52.9	nd	nd	nd	nd	nd	nd	nd
46	A	2,4-heptadienal[b,d]	17.36	971	green, oily, vegetable, cake, cinnamon	nd	nd	nd	nd	nd	46.91 ±49.6	nd	nd	nd	nd	nd	nd	nd	nd
47	A	octanal[b,e]	17.95	987	wax, citrus, orange peel, green, herbal, fresh	10.21 ±5.8	nd	189.91 ±352.5	812.11 ±114.3	734.32 ±469.2	143.54 ±47.2	1396.31 ±1518.7	77.24 ±41.0	73.62 ±56.8	239.37 ±59.3	150.42 ±54.0	nd	nd	nd
48	A	2-octenal[b,e]	20.40	1052	fresh, cucumber, green, herbal, banana, wax, green, leaf	nd	nd	26.05 ±14.4	136.31 ±12.6	180.46 ±80.5	162.36 ±40.0	118.13 ±87.2	nd	nd	nd	nd	nd	nd	nd
49	A	nonanal[b,e]	21.91	1092	wax, rose fresh, orange	40.12 ±25.9	240.81 ±289.9	351.16 ±121.3	2145.51 ±270.5	2451.38 ±1544.2	1352.68 ±400.5	4072.88 ±4027.0	484.48 ±258.2	266.99 ±159.0	1007.57 ±218.9	462.02 ±241.8	208.01 ±24.4	47.73 ±76.2	201.69 ±75.8

50	A	2-nonenal[b,f]	23.62	1138	green, cucumber, citrus	nd	nd	20.61 ±11.2	105.50 ±25.2	181.02 ±81.0	153.78 ±44.6	88.97 ±65.8	nd	5.25 ±5.4	64.20 ±69.7	27.86 ±28.7	nd	nd	nd
51	A	decanal[b,e]	25.58	1191	sweet, wax, orange peel, citrus, floral	111.81 ±114.8	nd	19.33 ±11.6	76.57 ±14.9	422.97 ±225.6	140.92 ±51.0	88.81 ±59.6	nd	16.56 ±17.1	42.47 ±39.6	45.78 ±32.6	nd	nd	nd
52	A	2-decenal[b,c]	26.93	1231	coriander, green, mushroom	nd	nd	nd	nd	99.98 ±139.5	nd	nd	nd	nd	nd	nd	nd	nd	nd
53	A	undecanal[b,e]	28.23	1270	wax, soap, floral, citrus, green, fresh laundry	nd	nd	nd	nd	62.21 ±30.6	26.70 ±3.6	nd	nd	nd	nd	nd	nd	nd	nd
54	A	tetradecanal[b,d]	36.17	1612	wax, amber, incense, dry, citrus peel, musk	nd	nd	nd	nd	nd	nd	nd	nd	nd	nd	nd	nd	184.12 ±105.8	nd
55	A	hexadecanal[b,d]	39.51	1819	cardboard	nd	nd	nd	nd	nd	nd	nd	nd	nd	nd	nd	nd	478.86±250.5	nd
56	O	isoamyl acetate[a,e]	13.15	858	sweet fruity, banana	nd	nd	nd	nd	nd	107.82 ±79.8	nd	nd	nd	nd	nd	nd	nd	78.84 ±76.4
57	O	methional[b,d]	14.24	887	musty potato, tomato, vegetable, creamy	nd	nd	nd	nd	nd	2795.63 ±20.35.3	nd	nd	nd	nd	nd	nd	nd	40.75 ±8.1
58	O	sulcatone[b,c]	16.45	947	citrus, green, lemongrass, apple	nd	nd	nd	nd	228.81 ±91.2	284.38 ±60.9	nd	nd	nd	nd	nd	nd	nd	nd
59	O	furan-2-pentyl[b,e]	17.20	967	fruit, green, bean, vegetable, metallic	185.82 ±154.4	219.40 ±123.3	193.96 ±59.3	230.93 ±42.7	151.65 ±46.3	482.68 ±144.9	133.69 ±62.7	36.03 ±18.1	32.06 ±17.0	nd	86.61 ±57.7	nd	nd	nd
60	O	hexyl acetate[a,e]	18.43	1000	fruity, green apple, banana sweet	nd	nd	nd	nd	nd	nd	nd	nd	14.96 ±17.4	nd	nd	nd	nd	188.81 ±217.0
61	P	methyl nicotinate[b,f]	21.72	1087	floral, rose dried, rose water	207.02 ±158.5	317.23 ±149.7	93.45 ±81.1	nd	nd	nd	nd	nd	92.60 ±71.5	nd	nd	222.72 ±244.7	nd	437.85 ±258.6

62	O	nonanoic acid[b,e]	26.64	1222	waxy, dirty cheese	nd	nd	nd	nd	nd	162.19 ±61.2	nd	nd	nd	nd	nd	nd	nd	nd
63	O	indole[b,e]	27.92	1261	animal floral, moth ball, fecal, naphthelene	nd	nd	404.59 ±590.3	nd	nd	nd	nd	nd	nd	nd	nd	nd	1393.13 ±1432.9	1113.55 ±759.5
64	O	α-Ionone[b,e]	29.70	1318	sweet, spicy, clove, woody	nd	nd	nd	nd	nd	nd	nd	nd	nd	nd	nd	nd	50.60 ±54.0	nd
65	O	trans-β-Ionone[b,d]	31.77	1397	sweet, fresh, spicy, clove, carnation, cinnamon	nd	nd	nd	nd	nd	nd	nd	nd	nd	nd	nd	nd	55.32 ±53.0	nd

# = compound number, nd = non detected, M = Monoterpene, S = Sesquiterpene, P = Phenylpropanoid, Al = Alcohol, A = Aldehyde, O = Other, **a** : efficient extraction with the first method(30 °C and 20 min), **b** : efficient extraction with the second method (75 °C and 65 min), **c** : identification performed by comparing the mass spectrum with that of the NIST library, **d** : identification performed by comparing the mass spectrum with that of the NIST library and by comparison of RI (retention index) with RI of, published literatures and online library (https://webbook.nist.gov/chemistry/cas-ser.html, accessed on 28 August 2021), **e** : identification performed by comparing the mass spectrum with that of the NIST library, by comparison of RI (retention index) with RI of published literatures and online library and by comparison of retention time and mass spectrum of the authentic standard, **f** : identification performed by comparing the mass spectrum with that of the NIST library and by comparison of retention time and mass spectrum of the authentic standard, **g** : odor characteristics were obtained from the "The Good Scents" company network database (www.thegoodscentscompany.com).
